# Frequency of Epstein-Barr virus DNA sequences in human gliomas: Frequência de sequências de DNA do vírus Epstein-Barr em gliomas humanos

**DOI:** 10.1590/1516-3180.2013.1912814

**Published:** 2014-11-28

**Authors:** Renata Fragelli Fonseca, Siane Lopes Bittencourt Rosas, José Antônio Oliveira, Anselmo Teixeira, Gilda Alves, Maria da Glória Costa Carvalho

**Affiliations:** I BSc, PhD. Postdoctoral Researcher, Congenital Malformations Laboratory, Department of Genetics, Universidade Federal do Rio de Janeiro (UFRJ), Rio de Janeiro, Brazil.; II BSc, PhD. Postdoctoral Researcher, Molecular Oncology Laboratory, Clementino Fraga Filho University Hospital, Universidade Federal do Rio de Janeiro (UFRJ), Rio de Janeiro, Brazil.; III MD. Neurosurgeon, Neurosurgery Service, Instituto Nacional de Câncer (INCA), Rio de Janeiro, Brazil.; IV PhD. Biologist, Instituto Nacional de Câncer (INCA), Rio de Janeiro, Brazil.; V MD, PhD. Professor, Molecular Pathology Laboratory, Pathology Department, Clementino Fraga Filho University Hospital, Universidade Federal do Rio de Janeiro (UFRJ), Rio de Janeiro, Brazil.

**Keywords:** Astrocytoma, Glioma, Brain neoplasms, Polymerase chain reaction, Herpesvirus 4, human

## Abstract

**CONTEXT AND OBJECTIVE:**

The Epstein-Barr virus (EBV) is the most common cause of infectious mononucleosis and is also associated with several human tumors, including Burkitt’s lymphoma, Hodgkin’s lymphoma, some cases of gastric carcinoma and nasopharyngeal carcinoma, among other neoplasms. The aim of this study was to screen 75 primary gliomas for the presence of specific EBV DNA sequences by means of the polymerase chain reaction (PCR), with confirmation by direct sequencing.

**DESIGN AND SETTING:**

Prevalence study on EBV molecular genetics at a molecular pathology laboratory in a university hospital and at an applied genetics laboratory in a national institution.

**METHODS:**

A total of 75 primary glioma biopsies and 6 others from other tumors from the central nervous system were obtained. The tissues were immediately frozen for subsequent DNA extraction by means of traditional methods using proteinase K digestion and extraction with a phenol-chloroform-isoamyl alcohol mixture. DNA was precipitated with ethanol, resuspended in buffer and stored. The PCRs were carried out using primers for amplification of the EBV BamM region. Positive and negative controls were added to each reaction. The PCR products were used for direct sequencing for confirmation.

**RESULTS:**

The viral sequences were positive in 11/75 (14.7%) of our samples.

**CONCLUSION:**

The prevalence of EBV DNA was 11/75 (14.7%) in our glioma collection. Further molecular and epidemiological studies are needed to establish the possible role played by EBV in the tumorigenesis of gliomas.

## RESUMO

**CONTEXTO E OBJETIVO:**

O vírus Epstein-Barr (EBV) é a causa mais comum de mononucleose infecciosa e também está associado com vários tumores humanos, tais como: linfoma de Burkitt, linfoma de Hodgkin, alguns casos de carcinoma gástrico e carcinoma nasofaríngeo, entre outras neoplasias. O objetivo deste estudo foi rastrear a presença de sequências de DNA-específicas do vírus EBV em biópsias de 75 gliomas primários usando reação em cadeia da polimerase (PCR), com confirmação por sequenciamento direto.

**TIPO DE ESTUDO E LOCAL:**

Estudo de prevalência sobre genética molecular de EBV em laboratório de patologia molecular de hospital universitário, e em laboratório de genética aplicada de instituição nacional.

**MÉTODOS:**

Foram obtidas 75 biópsias de gliomas primários e 6 de outros tumores do sistema nervoso central. Os tecidos foram imediatamente congelados para a extração posterior de DNA, através do método tradicional, usando a digestão por proteinase K e a extração pela mistura defenol-clorofórmio-álcool isolamílico. O DNA foi precipitado com etanol, ressuspendido em tampão e armazenado. As PCRs foram realizadas com iniciadores para amplificar a região BamM do EBV. Controles positivos e negativos foram adicionados a cada reação. Os produtos de PCR foram usados para sequenciamento direto para confirmação.

**RESULTADOS:**

As sequências virais foram positivas em 11/75 (14,7%) de nossas amostras.

**CONCLUSÃO:**

A prevalência de DNA de EBV foi de 11/75 (14.7%) na nossa coleção de gliomas. Mais estudos moleculares e epidemiológicos são necessários para esclarecer o possível papel do EBV na tumorigênese dos gliomas.

## INTRODUCTION

Little is known about the etiology of gliomas, although they are the most common histological type of tumor in the central nervous system (CNS). Recently, it was suggested that some virus families could be important contributors towards glioma development. Viruses may contribute towards human tumor development by inducing immunosuppression, modifying host cells through inducing oncoproteins, or altering the expression of host cell proteins at viral integration sites.

Herpes viruses can infect humans easily. The timing of infection is related to living conditions, and several members of the herpes family possess known transformational properties, notably the Epstein-Barr virus (EBV). About 90% of the world’s population is estimated to be infected by EBV. Primary EBV infection is spread mainly through saliva transfer between individuals. Following primary infection, which may be either symptomatic or silent, this virus has two distinct life cycles in the human host: a lytic cycle, during which the production of new virions occurs; and a latent form, which remains in the host.^1^

EBV is associated with several human malignancies including Burkitt’s lymphoma, Hodgkin’s disease, nasopharyngeal carcinoma (NPC), peripheral T-cell lymphoma, thymoma and gastric cancer.^2^^,^^3^^,^^4^^,^^5^

EBV has been intensely studied, not only because of its ability to cause lifelong persistent infection but also because it is causally associated with a number of diseases in the CNS (infectious mononucleosis, demyelinating disease, acute encephalitis, acute cerebellar ataxia, myelitis or meningitis).^6^ EBV is thought to be responsible for a number of neurological syndromes, such as diffuse or focal encephalitis, aseptic meningitis, Guillain-Barré syndrome and peripheral neuropathy, among others.^7^^,^^8^ It has been demonstrated that astrocyte cell lines and human fetal astrocytes are the only brain cells that express complement receptor type 2 (CR2), the major cellular receptor for EBV.^9^ EBV has been found to be able to infect astrocyte cell lines.^10^ These findings together support the idea that EBV could act as an etiological agent in brain diseases.

## OBJECTIVE

The aim of this study was to screen 75 primary astrocytomas for the presence of specific EBV DNA sequences, by means of the polymerase chain reaction (PCR), with confirmation using direct sequencing.

## METHODS

### Patients and tumor samples

Between 1997 and 2001, a total of 75 primary glioma biopsies and six others from other tumors from the CNS were obtained during surgery performed by the neurosurgery service at a cancer treatment institution in the city of Rio de Janeiro, Brazil. This work was approved by the institution’s ethics board, under registration number 35/02. The patients’ participation was voluntary and their data was confidential. To confirm the participation, the volunteer signed a clear statement of informed consent, after having been informed of all aspects of the study. When the patient was underage, his/her parents were asked to sign the informed consent. The number of patients recruited was based on the number of new registrations in the Neurosurgery Service between the years 1997 and 2001.

The tissues were immediately snap-frozen in liquid nitrogen, and subsequently stored at - 70 °C until deoxyribonucleic acid (DNA) extraction. Histological diagnoses were first made on the specimens during surgery and were later on confirmed by the pathology service of the same institution. These patients had no clinical evidence demonstrating that the tumors generated metastasis. We followed the World Health Organization (WHO) brain tumor classification.

### DNA extraction

The samples were subjected to proteinase K digestion (100 mg/ml) in the presence of 0.5% SDS at 37 °C overnight. This was followed by phenol-chloroform-isoamyl alcohol (25:24:1) extraction and ethanol precipitation. The DNA was resuspended in buffer and stored at -20 °C until molecular analysis was performed.^11^

### Polymerase chain reaction

The PCRs were carried out in a final volume of 25 ml containing 100 ng of DNA, 1 mM of each primer, 10 mM of Tris-HCl (pH 8.3), 50 mM of KCl, 1.5 mM of MgCl_2_, 200 µM of each nucleotide and 0.125 U of Taq polymerase. The primers were selected so as to amplify a 288 bp DNA product from the EBV BamM region: CAGGCTTCCCTGCAATTTTACAAGCGG and CCCAGAAGTATACGTGGTGACGTAGA.^12^ A negative DNA-free control and a positive control from the EBV-positive Raji cell lineage were included in each assay.

Thermal cycling was performed using the following conditions: initial denaturation at 95 °C for five minutes; 40 cycles of ramping to 94 °C for one minute; cooling to 55 °C for two minutes; heating to 72 °C for one minute; and a final extension step at 72 °C for seven minutes. PCR fragments were separated by means of electrophoresis on 8% PAGE gel in 1X TBE buffer, co-migrating with a 100 bp ladder marker, and this was followed by silver staining as previously described.^13^ As an internal control for DNA quality, we used primers to amplify exon 5 of the tumor suppressor gene TP53: GCAACCAGCCCTGTCGTGTCTCCA and GAATTCTGTTCACTTGTGCCCTGACTTTCAAC.^14^ All the samples analyzed for the presence of EBV amplified the suppressor gene TP53.

### Sequencing reaction

The PCR product was purified using the GFX PCR DNA gel band purification kit (GE Healthcare Biosciences, Buckinghamshire, UK), in accordance with the manufacturer’s instructions, and this was used for direct sequencing in an ABI 3130 device (Life Technologies, CA, USA). The alignments were obtained through the GenBank online Blast-N software, which is available from the National Center for Biotechnology Information (http://www.ncbi.nlm.nih.gov).

## RESULTS

The glioma specimens were classified as astrocytoma grade I (7/75), astrocytoma grade II (25/75), astrocytoma grade III (16/75), glioblastoma multiforme (18/75), ependymoma (5/75), oligodendroglioma (2/75) and oligoastrocytoma (2/75). These tumors were from 40 males and 35 females; 25 patients were children (under 21 years of age) and 50 were adults. The location of the tumors in the brain was variable: temporal lobe, fourth ventricle, thalamus, cerebellar vermis, brain stem, cerebral ventricle, supra tentorial, occipital lobe and parietal lobe.

The DNA extracted from the gliomas was screened for the presence of EBV DNA. The result was positive in 11/75 (14.7%) of the tumor specimens. The tumors positive for EBV DNA were: astrocytoma grade II (6/11); astrocytoma grade III (2/11); oligoastrocytoma (1/11); ependymoma (1/11); and glioblastoma multiforme (1/11) (Table 1).

**Table 1.**
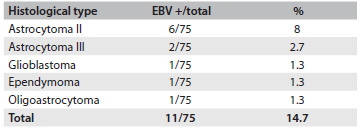
Histological subtype and number of samples positive for Epstein-Barr virus (EBV) deoxyribonucleic acid (DNA)

**Figure 1.**
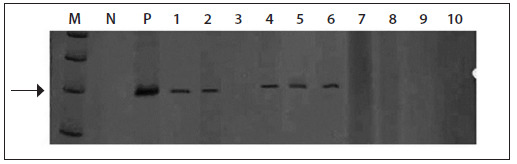
Silver-stained 8% PAGE gel showing examples of polymerase chain reaction (PCR) products from the Epstein-Barr virus (EBV), from glioma biopsies.

Figure 1 shows a gel containing positive PCR reactions. Additionally, the presence of EBV DNA was also screened for in other CNS tumors (one neurilemma, two non-Hodgkin lymphomas, two pituitary adenomas and one cortical dysplasia) and they were all negative for EBV DNA.

The homology between the EBV sequences found in the astrocytoma patients was compared with the published EBV sequence, by examining their similarity. The identicalness rate observed (95.5%) confirmed that the virus found was EBV.

## DISCUSSION

Over the past decade, several groups have examined the evidence for the presence and expression of viruses in CNS tumors and have analyzed the quantity of viral material. Reports on SV40 (simian virus 40), JCV (John Cunningham virus) and HCMV (human cytomegalovirus) have been made, with diverse findings.^15^^,^^16^^,^^17^^,^^18^^,^^19^^,^^20^^,^^21^^,^^22^^,^^23^^,^^24^ More recently, JCV and HCMV have been accepted as being associated with CNS tumors.^25^ Discordant findings may occur because of the sensitivity of the PCR/*in situ* techniques used, difficulty in working with paraffin-embedded tissues and also small numbers of samples. In the present study, it is important to note that we used 75 fresh frozen tissue samples and conventional PCR, which has the advantage of amplifying small target sequences. In this study, in order to search for EBV DNA in different histological types/grades (Table 1), we used the simple method of conventional PCR, which had been used in previous work.^12^ The prevalence of EBV DNA found was 11/75 (14.7%).

There is evidence showing that EBV emerges from latency in immunosuppressed individuals. Many cancer patients present immunosuppression, which may be caused by aging, by the disease itself or even by medications, and EBV and other silent viruses may be activated in this manner.^26^^,^^27^ The EBV genome is complex and comprises at least 12 known genes; however, the most famous EBV transforming protein is LMP1 (latent-infection membrane protein 1).^28^ In spite of all of this knowledge, the role of EBV in glioma tumorigenesis is still not understood.

An EBV vaccine has been tested (in a phase I clinical trial) on Chinese nasopharyngeal carcinoma patients to determine the safe and immunogenic dose.^29^ In that study, it was concluded that the vaccine is both safe and immunogenic, thus allowing the highest dose to be moved forward to phase II studies. From our data, it is possible that in the future, in the same way as seen among those nasopharyngeal carcinoma patients, tests using an EBV vaccine may be found to benefit glioma patients.

## CONCLUSION

The prevalence of EBV DNA was 11/75 (14.7%) in our glioma samples. Further molecular and epidemiological studies are needed in order to establish the possible role played by EBV in the tumorigenesis of gliomas.
